# An Evaluation of the Pea Pod System for Assessing Body Composition of Moderately Premature Infants

**DOI:** 10.3390/nu8040238

**Published:** 2016-04-22

**Authors:** Elisabet Forsum, Elisabeth Olhager, Caroline Törnqvist

**Affiliations:** 1 Department of Clinical and Experimental Medicine, Linköping University, SE-581 83 Linköping, Sweden; caroline.tornqvist@regionostergotland.se; 2 Department of Clinical Sciences, Lund University, SE-221 85 Lund, Sweden; elisabeth.olhager@skane.se

**Keywords:** body composition, fat-free mass density, isotope dilution, Pea Pod, premature infants, three-component model

## Abstract

(1) Background: Assessing the quality of growth in premature infants is important in order to be able to provide them with optimal nutrition. The Pea Pod device, based on air displacement plethysmography, is able to assess body composition of infants. However, this method has not been sufficiently evaluated in premature infants; (2) Methods: In 14 infants in an age range of 3–7 days, born after 32–35 completed weeks of gestation, body weight, body volume, fat-free mass density (predicted by the Pea Pod software), and total body water (isotope dilution) were assessed. Reference estimates of fat-free mass density and body composition were obtained using a three-component model; (3) Results: Fat-free mass density values, predicted using Pea Pod, were biased but not significantly (*p* > 0.05) different from reference estimates. Body fat (%), assessed using Pea Pod, was not significantly different from reference estimates. The biological variability of fat-free mass density was 0.55% of the average value (1.0627 g/mL); (4) Conclusion: The results indicate that the Pea Pod system is accurate for groups of newborn, moderately premature infants. However, more studies where this system is used for premature infants are needed, and we provide suggestions regarding how to develop this area.

## 1. Introduction

The quality of growth of premature infants is an important topic which has been studied for decades. Even several years ago, it had been pointed out that “weight gain” by itself is not sufficient as a parameter when growth of infants is studied and that measuring changes in body composition is the way to obtain information regarding the impact of nutrition on growth [1]. It is often considered that premature infants should gain weight at a rate comparable to the intrauterine growth rate, *i.e.*, their weight gain should be the same as if they had remained *in utero* until full-term gestational age [2]. However, recent studies tend to show that the weight gained after birth by preterm infants consists of a comparatively large proportion of fat [3]. Such data have been obtained by means of the Pea Pod device, using a procedure shown to be accurate in full-term infants [4]. Roggero *et al.* demonstrated that this device can produce reproducible estimates of body composition in premature infants and reported a validation study conducted in ten such infants, 15 days old on average [5]. These authors used a two-component model based on total body water to calculate reference estimates of body composition and concluded that the amount of body fat, assessed using Pea Pod, is in good agreement with such reference estimates. However, due to incomplete information regarding the hydration and density of fat-free mass in premature infants, it has been recommended that their body fat content should be assessed using three- or four-component models [6] (p. 26). Therefore, knowledge regarding the body composition of newborn premature infants is still limited, and this area requires further studies [7].

The Pea Pod system is based on the two-component model [8] and calculates body fat from body weight and volume. This requires appropriate figures for the density of fat and fat-free mass. The density of body fat is generally considered to be constant at 0.9007 g/mL, while the density of fat-free mass varies with age and physiological status. For premature infants, the Pea Pod procedure applies figures for fat-free mass density obtained using back-extrapolation [9] of data for full-term infants [10]. However, these extrapolated figures have not been experimentally confirmed. In the present paper, we assessed fat-free mass density of moderately premature infants during the first week of life using a three-component model based on total body water, body weight, and body volume and compared the results to the corresponding fat-free mass density predicted by the Pea Pod software. We also compared values for body fat, obtained using the three-component model, to body fat assessed using Pea Pod.

## 2. Materials and Methods

### 2.1. Infants

Parents of infants, without sepsis or malformations and born after at least 32 but less than 37 completed gestational weeks, were asked for permission to allow their child to participate in the study. Both parents of 14 infants agreed to participate. None of these infants needed ventilatory support, extra oxygen, or parenteral nutrition. The reasons for premature birth were high maternal blood pressure, bleeding or infection, premature rupture of membranes, or unknown. At the time of investigation, infants were fed their own mother’s milk and/or human milk from a bank. Some of them were also fed small amounts of formula for premature infants. All infants had passed their initial weight loss and had started to gain weight. The regional human research ethics committee in Linköping approved the study (2011/313-32; M128-04) on 21 September 2011. Parents of all infants gave informed consent.

### 2.2. Protocol

Two urine samples were collected from the infant on the day before the study. The following morning body weight and volume were measured and body fat calculated, using Pea Pod (COSMED USA, Inc., Concord, CA, USA). Thereafter, a dose of stable isotopes (0.08 g ^2^H_2_O and 0.16 g H_2_^18^O per kg body weight) was administered to the infant through a naso-gastric tube. The exact dose given was determined by weighing the syringe on an analytical balance before and after dose administration. The naso-gastric tube was flushed with saline after dosing. The time of dosing was noted. Before dosing, cotton balls were placed in the diapers, and the amount of all urine produced during 6–10 h after dosing was measured by weighing diapers with these balls before and after use. To obtain urine specimens, cotton balls were placed in a syringe, and the urine was squeezed into glass vials with an internal aluminum-lined screw cap sealing and stored at −20 °C.

### 2.3. Total Body Water

Urine samples were analyzed using an isotope ratio mass spectrometer (Deltaplus XL, Thermoquest, Bremen, Germany) as previously described [11]. Deuterium space and oxygen-18 space were calculated using the maximum isotope enrichments in urine which occurred 6.5 ± 1.2 (range: 5.8–9.5) h after dosing (plateau-method) [12]. The amount of isotope excreted in urine before maximum enrichment was deducted from the dose when calculating dilution spaces. Total body water was calculated as the mean value of deuterium space/1.041 and oxygen-18 space/1.007 [6] (p. 34).

### 2.4. Pea Pod

As previously described [13], the procedure started with a measurement of the length of the infant using a length board and was followed by a recording of the body weight using a scale, which is part of the Pea Pod. Then, the infant was placed without clothing in the Pea Pod chamber for about two minutes to measure body volume using air displacement plethysmography. The infant’s hair was kept flat by means of a tight cap when volume was measured. Adjustments were made for the weight and volume of any articles not removable from the infant (*i.e.*, naso-gastric tube, identification tape). All infants were examined by the same person (C.T.). None of them cried excessively during the measurement. The measurements were conducted using Pea Pod software 3.0.1 (Cosmed, Concord, CA, USA, which also calculates body density from body weight, body volume, and subsequently body composition based on the fat-free mass density values predicted via the software.

### 2.5. Fat-Free Mass Density and Hydration and Body Fat Calculated Using a Three-Component Model

Fat-free mass density and the fraction of fat in the body (f) were calculated using the following two equations:

f = [(2.028/body density) − (0.789 × total body water/body weight) − 1.2538](1)

1/body density = f/0.9007 + (1 − f)/fat-free mass density(2)

Equation (1) represents the three-component model developed by Siri [14], which requires an assumption regarding the mineral–protein ratio in the body. In the common version of this model, this ratio is 0.35, which is considered to be appropriate for adults [14]. In the present paper, we assume this ratio to be 0.45, which is based on data for fetuses weighing 1500–3000 g [15]. This ratio was used in Equation (1), and this equation is therefore appropriate for premature infants and also slightly different from the adult version of this model [14]. Equation (2) represents the two-component model [8]. Hydration of fat-free mass, the so-called “hydration factor”, was calculated as [total body water (g)/fat-free mass (g)] × 100.

### 2.6. Biological Variability of Fat-Free Mass Density

Variability was expressed as standard deviation (SD) and calculated using the following equation [11]: (SD_t_)^2^ = (SD_m_)^2^ + (SD_b_)^2^, where SD_t_ is the total variability of the fat-free mass density, and SD_m_ the appropriate methodological error. We assumed SD_m_ to be uncorrelated to the biological variability (SD_b_) and 0.00126 g/mL, as previously estimated in full-term healthy neonates [11].

### 2.7. Statistics

Values given are means and SDs. Student’s *t*-test and linear regression analysis were used; *p* < 0.05 was statistically significant. All hypothesis tests were two-sided. Methods were compared according to Bland–Altman [16]. Statistica 10 (Statasoft, Scandinavia AB, Uppsala, Sweden) was used for statistical analyses.

## 3. Results

The infants (10 boys and 4 girls) in the study are described in Table 1. They were born after 32 to 35 completed gestational weeks and were 3–7 days old when investigated. At birth, their average weight and length *z* scores were both close to −1. Table 2 shows their body weight and volume, total body water, and body density on the day of investigation.

Table 3 shows that average values for fat-free mass density and body fat (%) produced by the Pea Pod system are slightly but not significantly higher than the corresponding values obtained using the three-component model. The density of fat-free mass was 1.0627 ± 0.0059 g/mL when based on the three-component model. Using this SD (0.0059 g/mL), we found the biological variability of the fat-free mass density to be 0.0058 g/mL equivalent to 0.55% of its average value. Table 3 also shows that the hydration factor is 83% when based on the three-component model. It is relevant to compare this value to published values [15] for the hydration factor of fetuses with a gestational age similar to that of our infants. We estimated this figure to be 84.3%, on average, which is comparable to the figure (83.0%) in Table 3.

**Figure 1:**
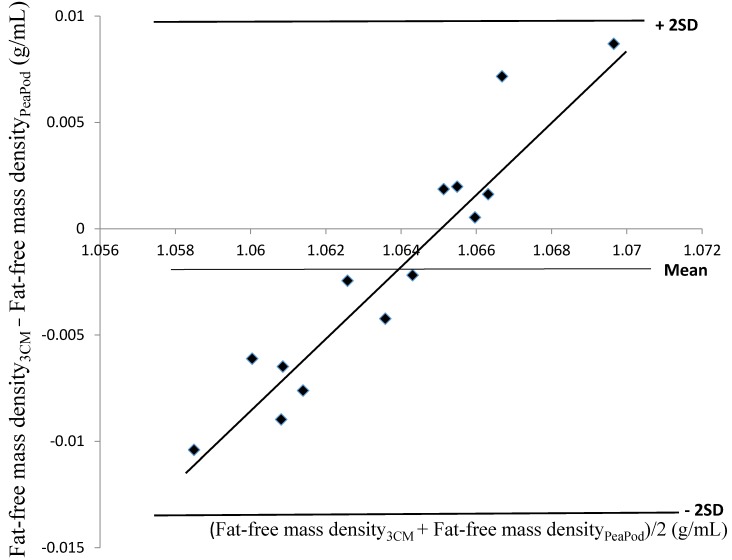
The difference between fat-free mass density (g/mL), assessed by means of the three-component model (Fat-free mass density_3CM_) and the corresponding values predicted by the Pea Pod software (Fat-free mass density_PeaPod_) (Fat-free mass density_3CM_ − Fat-free mass density_PeaPod_) (*y*) versus the average of Fat-free mass density_3CM_ and Fat-free mass density_PeaPod_ (Fat-free mass density_3CM_ + Fat-free mass density_PeaPod_)/2 (*x*). Average and ± 2SD (limits of agreement) for (Fat-free mass density_3CM_ − Fat-free mass density_PeaPod_) = −0.0019 ± 0.0117 g/mL. Regression equation: *y* = 1.79*x* − 1.90, *r*^2^ = 0.91 (*p* < 0.001). Values are based on 14 moderately premature infants, 3–7 days old.

**Figure 2:**
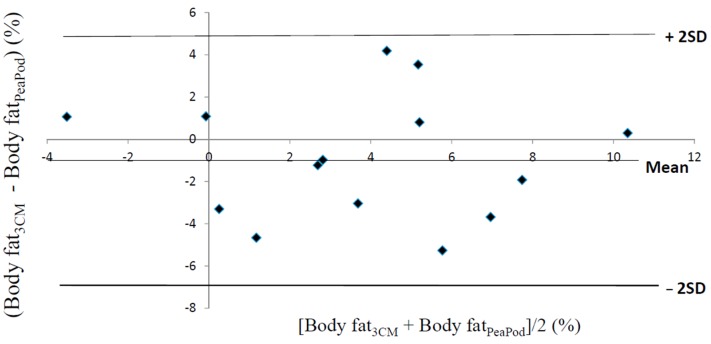
The difference between body fat, in % of body weight, assessed by means of the three-component model (Body fat_3CM_) and the corresponding values assessed by means of the Pea Pod system (Body fat_PeaPod_) (Body fat_3CM_ − Body fat_PeaPod_) (*y*) versus the average of Body fat_3CM_ and Body fat_PeaPod_ (Body fat_3CM_ + Body fat_PeaPod_)/2 (*x*). Average and ± 2SD (limits of agreement) for Body fat_3CM_ − Body fat_PeaPod_ = −1.00% ± 5.83%. Values are based on 14 moderately preterm infants, 3–7 days old.

Fat-free mass density values, predicted by the Pea Pod software, were not correlated with fat-free mass density values assessed using the three-component model (*r*^2^ = 0.04, *p* > 0.05). These values were 0.0019 ± 0.0058 g/mL lower (*p* > 0.05) than the corresponding values predicted by the Pea Pod software. A Bland–Altman evaluation of these results is shown in Figure 1. The limits of agreement were −0.0136 → +0.0098 g/mL. The relationship between the difference between the two methods (*y*) and their average (*x*) was significant (*r*^2^ = 0.91, *p* < 0.001). In contrast, estimates of body fat (%), obtained by means of Pea Pod, were significantly correlated (*r*^2^ = 0.51, *p* < 0.01) with body fat (%) assessed using the three-component model. Figure 2 shows a Bland–Altman evaluation for body fat (%). Body fat assessed using the three-component model was 1.00 ± 2.91 percentage points (*p* > 0.05) lower than the corresponding values assessed using the Pea Pod system. The limits of agreement were −6.8% → +4.8% body fat. No significant relationship (*r*^2^ = 0.0013, *p* > 0.05) was present between the difference between the two methods (*y*) and their average (*x*).

## 4. Discussion

It should be noted that the infants in this study had comparatively low *z* scores for length as well as for weight, indicating that they may not necessarily be representative of infants with a more average size. Nevertheless, due to the medical background of their premature birth and their clinical history after birth, it is reasonable to regard them as a group of healthy premature infants. The strengths of this study are that the Pea Pod results were evaluated using results obtained by means of a three-component model, and that it is the first validation study conducted in moderately premature infants during the first week of life and the first to provide estimates of fat-free mass density and variability in premature infants. A limitation is the small size of the study. However, the reason for this is the very limited availability of eligible subjects, a fact explaining the lack of published validation studies conducted in premature newborn infants.

It is important to point out that our evaluation assumes that air displacement plethysmography, as applied in the Pea Pod system, is able to assess body volume accurately. This assumption is based on data showing that this system measures volume of inanimate objects with high accuracy [18]. However, several of our subjects had a volume smaller than 1.85 L, which is the smallest test volume used in that study [18]. Furthermore, Pea Pod corrects the measured volume using predicted values for thoracic gas volume and surface area artifacts [18], corrections which may introduce errors. Pea Pod has been reported to be accurate in full-term infants [4], indirectly supporting the statement that these corrections are accurate for such infants. It has also been reported that small premature infants can be measured in Pea Pod with good reproducibility [5]. Nevertheless, it is conceivable that accuracy may be impaired when the volume of very small infants is measured in Pea Pod; further studies on this issue are encouraged.

In this study, body water was assessed using the plateau-procedure [12] which requires that the amount of isotope excreted during the equilibration period is deducted from the dose. We measured the amount of dose excreted in urine but not the dose lost via the insensible water losses. Using values for such losses estimated in premature infants [19] we found that the amount of isotope lost through this route is too small to affect our results in any important way.

Furthermore, body water was measured six to ten hours after the measurements of body volume and body weight were taken in Pea Pod. Therefore, since our infants had started to gain weight and consequently body water, their body water content tends to be slightly overestimated. Based on values for their weight gain, we calculated that the average difference between the Pea Pod and reference estimates was overestimated by about 10%. Thus, the difference in fat-free mass density would have been 0.0017 rather than 0.0019 g/mL, and the corresponding difference in body fat 0.9% rather than 1%. Consequently, we conclude that our study did not identify any significant differences between Pea Pod data and reference estimates of fat-free mass density or body fat; moreover, we also concluded that, if there are such differences, they are likely to be small. The difference in timing for body water and Pea Pod measurements had only marginal effects on all other results presented in this paper.

Our paper shows that predictions of fat-free mass density by the Pea Pod software were, on average, in reasonable agreement with reference average estimates. Furthermore, reference estimates of the hydration factor were, on average, comparable to a corresponding estimate based on published data [15]. The water content of fat-free mass is a main determinant of its density; therefore, our findings support the conclusion that published information regarding fat-free mass composition of fetuses in late gestation is reasonably correct on average.

The data presented in Figure 1 represent the first evaluation of Pea Pod predicted values for fat-free mass density of premature infants *versus* reference estimates. This figure clearly shows that such Pea Pod values suffer from a bias by which a low fat-free mass density is overestimated, while a high such value is underestimated. Furthermore, we found no correlation between fat-free mass density assessed using Pea Pod and the corresponding figures assessed using the three-component model. These observations question the capacity of the Pea Pod software to predict the fat-free mass density of premature infants. Fortunately, when body fat was calculated using fat-free mass density predicted using the Pea Pod software, no bias was detected in the Bland–Altman evaluation. Nevertheless, the bias and lack of correlation found for fat-free mass density values represent a concern. However, considering the limited information available regarding body composition of premature infants, it is presently very difficult to improve the equations used by Pea Pod to predict the fat-free mass density of such infants. Consequently, further studies of factors influencing the body composition of premature infants are recommended.

In spite of the problems discussed above, our evaluation supports the conclusion that Pea Pod is able to produce valid average estimates of body fat in a group of premature newborn infants. However, the large limits of agreement shown in Figure 2 (−6.8% → +4.8% body fat) suggest that estimates for individual infants may deviate considerably from reference values. This is not surprising considering the limited capacity of Pea Pod to predict the fat-free mass density of individual infants. However, in this context, it is relevant to consider results obtained in other studies. For example, in their study on the accuracy of air displacement plethysmography for full-term infants, Ellis *et al.* found the limits of agreement to be −6.8% → +8.1% body fat when estimates obtained using the Pea Pod system were compared to reference estimates obtained by means of a four-component model [4]; in a study on adult women, such values were approximately −8% → +4% body fat [20]. In the latter study [20], the aqueous fraction of fat-free mass was reported to have a significant effect on the estimates of body fat obtained using air displacement plethysmography and the two-component model. Therefore, we considered it relevant to assess the biological variability of fat-free mass density in our premature newborn infants. We found this variability to be 0.55% of the average fat-free mass density, a figure comparable to the corresponding figure, 0.64%, reported for adults [21]. The water content of fat-free mass, an important determinant of its density, decreases during fetal life. This introduces a variation in fat-free mass density which is included in our estimate of its biological variability. This source of variation is to some extent taken into account when premature infants are measured in Pea Pod since calculations are then based on fat-free mass density values which are specific for the stage of gestation. Therefore, the biological variability of fat-free mass density relevant when moderately premature newborn infants are measured in Pea Pod is likely to be lower than 0.55% of the average fat-free mass density and thus at least as good as the corresponding variability in adults. This suggests that a procedure to assess body fat based on air displacement plethysmography and the two-component model has the potential to be as valid in premature infants as it is in adults.

When infants with a low body fat content, such as ours, are measured in Pea Pod, a negative body fat content may be found. Obviously, this is not physiologically plausible, but may occur as a result of measurement errors. Such errors may be present in the assessments of volume or weight, and the value for fat-free mass density used in the Pea Pod system may differ from the true such value of the subject under study. These errors are not necessarily higher for premature infants than for older subjects but are more likely to result in negative values when subjects with a low body fat content are studied. When adult subjects are studied in a similar way, corresponding errors will not result in a negative body fat content, since such subjects contain much more body fat than newborn premature infants. In this context, it is relevant to note that, when a group of infants is studied, excluding results showing a body fat content below 0% will lead to a biased estimate for the group. However, as pointed out above, knowledge regarding the body composition of premature infants is still limited; it is therefore possible that other, as yet unknown factors are responsible for the negative values sometimes observed when body fat of young infants is assessed by means of Pea Pod.

## 5. Conclusions

This study shows that estimates of fat-free mass density were biased and that individual estimates of body fat may have deviated considerably from reference values when the Pea Pod system was applied to moderately premature newborn infants. Nevertheless, this system was found to deliver satisfactory average values for the body fat and fat-free mass density of moderately premature newborn infants, and our evaluation supports the conclusion that Pea Pod estimates of body fat are accurate for groups of such infants. However, due to the limitations discussed above, more studies of the accuracy of the Pea Pod system for moderately premature newborn infants are needed, and our paper provides suggestions regarding how this area can be developed.

## Figures and Tables

**Table 1:** Characteristics of infants in the study.

Sex	Gestational Age at Birth Weeks + Days	Age at Investigation Days	Weight at Birth g	Weight *z* Score ^1^	Length at Birth cm	Length *z* Score ^1^
male	33 + 1	7	1710	−1.93	43	−2.29
male	35 + 3	5	2090	−1.77	45	−1.33
male	33 + 6	4	2306	0.34	45	−0.60
male	33 + 0	6	2370	0.56	46	0.85
female ^2^	32 + 4	6	1805	−1.20	41	−2.36
female ^2^	32 + 4	6	1780	−1.30	40	−3.07
male	35 + 6	4	2685	−0.44	48	0
male	32 + 5	7	1970	−0.85	43	−1.29
male ^3^	35 + 0	4	1875	−2.60	45	−1.33
male ^3^	35 + 0	4	1750	−3.14	45	−1.33
male	35 + 2	3	2520	−0.31	47	0
female	35 + 2	3	2510	−0.04	44	−1.64
female	35 + 4	4	2680	−0.14	47	−0.20
male	32 + 3	5	1790	−0.80	44	0.21
mean	34 + 1	4.9	2132	−1.02	45	−0.96
SD	1 + 2	1.4	367	1.04	2	1.06

^1^ Calculated using Swedish reference data [17]; ^2^ monochorionic twins; ^3^ twins.

**Table 2:** Body weight, body volume, total body water and body density of 14 moderately premature infants at the age of 3–7 days.

	Mean ± SD
Body weight ^1^, kg	2.038 ± 0.332
Body volume ^2^, L	1.930 ± 0.322
Total body water ^3^, kg	1.630 ± 0.240
Total body water ^3^, %	80.30 ± 3.87
Body density ^4^, g/mL	1.057 ± 0.007

^1^ Assessed using the scale in Pea Pod; ^2^ assessed by means of air displacement plethysmography in Pea Pod; ^3^ assessed using isotope dilution; ^4^ calculated using Pea Pod software 3.0.1 from body weight and body volume.

**Table 3:** Fat-free mass density and body fat of 14 moderately premature infants, 3–7 days old, assessed using the Pea Pod system or using a three-component model. The hydration factor is also shown.

	Fat-Free Mass Density (g/mL)	Body Fat (%)	Hydration Factor (%)
Pea Pod system	1.0646 ± 0.0009	4.2 ± 3.9	-
Three-component model	1.0627 ± 0.0059 ^1^	3.2 ± 3.8 ^1^	83.0 ± 1.4

^1^ Not significantly different (*p* > 0.05) from the corresponding value assessed using the Pea Pod system.

## Abbreviations

The following abbreviations are used in this paper:

3CM: Three-component model
f: fraction of fat in the bodyl
